# Molecular Analysis of *bla*_KPC-2_-Harboring Plasmids: Tn*4401a* Interplasmid Transposition and Tn*4401a*-Carrying ColRNAI Plasmid Mobilization from Klebsiella pneumoniae to Citrobacter europaeus and Morganella morganii in a Single Patient

**DOI:** 10.1128/mSphere.00850-21

**Published:** 2021-11-03

**Authors:** Kayoko Sugita, Kotaro Aoki, Kohji Komori, Tatsuya Nagasawa, Yoshikazu Ishii, Satoshi Iwata, Kazuhiro Tateda

**Affiliations:** a Department of Microbiology and Infectious Diseases, Toho University Graduate School of Medicine, Tokyo, Japan; b Department of Microbiology and Infectious Diseases, Toho University School of Medicine, Tokyo, Japan; c Department of Microbiology and Immunology, Keio University School of Medicine, Tokyo, Japan; d Department of Infectious Diseases, Keio University School of Medicine, Tokyo, Japan; e Department of Infectious Diseases, National Cancer Center Hospital, Tokyo, Japan; JMI Laboratories

**Keywords:** KPC-producing organisms, plasmid, Tn*4401a*, *bla*_KPC-2_, WGS

## Abstract

The spread of Klebsiella pneumoniae carbapenemase (KPC)-producing *Enterobacterales* is a public health concern. KPC-encoding *bla*_KPC_ is predominantly spread by strains of a particular phylogenetic lineage, clonal group 258, but can also be spread by horizontal transfer of *bla*_KPC_-carrying plasmids. Here, we report the transfer of a *bla*_KPC-2_-harboring plasmid via mobilization from K. pneumoniae to Citrobacter freundii complex and Morganella morganii strains in a single patient. We performed draft whole-genome sequencing to analyze 20 carbapenemase-producing *Enterobacterales* strains (15 of K. pneumoniae, two of C. freundii complex, and three of M. morganii) and all K. pneumoniae strains using MiSeq and/or MinION isolated from a patient who was hospitalized in New York and Montreal before returning to Japan. All strains harbored *bla*_KPC-2_-containing Tn*4401a*. The 15 K. pneumoniae strains each belonged to sequence type 258 and harbored a Tn*4401a*-carrying multireplicon-type plasmid, IncN and IncR (IncN+R). Three of these K. pneumoniae strains also possessed a Tn*4401a*-carrying ColRNAI plasmid, suggesting that Tn*4401a* underwent interplasmid transposition. Of these three ColRNAI plasmids, two and one were identical to plasmids harbored by two Citrobacter europaeus and three M. morganii strains, respectively. The Tn*4401a*-carrying ColRNAI plasmids were each 23,753 bp long and incapable of conjugal transfer via their own genes alone, but they mobilized during the conjugal transfer of Tn*4401a*-carrying IncN+R plasmids in K. pneumoniae. Interplasmid transposition of Tn*4401a* from an IncN+R plasmid to a ColRNAI plasmid in K. pneumoniae and mobilization of Tn*4401a*-carrying ColRNAI plasmids contributed to the acquisition of *bla*_KPC-2_ in *C. europaeus* and M. morganii.

**IMPORTANCE** Plasmid transfer plays an important role in the interspecies spread of carbapenemase genes, including the Klebsiella pneumoniae carbapenemase (KPC)-coding gene, *bla*_KPC_. We conducted whole-genome sequencing (WGS) analysis and transmission experiments to analyze *bla*_KPC-2_-carrying mobile genetic elements (MGEs) between the *bla*_KPC-2_-harboring K. pneumoniae, Citrobacter europaeus, and Morganella morganii strains isolated from a single patient. *bla*_KPC-2_ was contained within an MGE, Tn*4401a*. WGS of *bla*_KPC-2_-carrying K. pneumoniae, *C. europaeus*, and M. morganii strains isolated from one patient revealed that Tn*4401a*-carrying ColRNAI plasmids were generated by plasmid-to-plasmid transfer of Tn*4401a* from a multireplicon-type IncN and IncR (IncN+R) plasmid in K. pneumoniae strains. Tn*4401a*-carrying ColRNAI plasmids were incapable of conjugal transfer in *C. europaeus* and M. morganii but mobilized from K. pneumoniae to a recipient Escherichia coli strain during the conjugal transfer of Tn*4401a*-carrying IncN+R plasmid. Therefore, Tn*4401a*-carrying ColRNAI plasmids contributed to the acquisition of *bla*_KPC-2_ in *C. europaeus* and M. morganii.

## INTRODUCTION

The spread of carbapenemase-producing *Enterobacterales* (CPE) is a public health concern (1). Klebsiella pneumoniae carbapenemase (KPC) genes (*bla*_KPC_) have now spread worldwide and are particularly prevalent in North and South America, China, Greece, and Israel (1). K. pneumoniae strains belonging to clonal group 258 (CG258), including those of sequence type 11 (ST11), ST258, and ST512, are associated with the spread of *bla*_KPC_ (2–8).

The Tn*3*-family transposon Tn*4401* is an important mobile genetic element for carrying *bla*_KPC_ (9–11). To date, nine Tn*4401* isoforms have been reported (12–17). The IncF group pKpQIL-like plasmid carrying Tn*4401* has been analyzed in detail (18–21). Furthermore, *bla*_KPC_-carrying Inc plasmids, e.g., IncFIIK1, IncFIIK2, IncFIA, IncN, IncI2, IncX, IncA/C, IncR, and ColE1, have been detected in various ST258 clones (5, 18, 22).

Interstrain and -species horizontal transfer of genes encoding carbapenemases is important for the spread of CPE (23, 24). *bla*_KPC_-carrying plasmids can be transmitted to other strains or species, resulting in the emergence of new KPC-producing strains (25). In our case study, KPC-type CPE, which are rarely detected in Japan (1), were detected in one patient comprising not only *bla*_KPC_-positive K. pneumoniae but also *bla*_KPC_-positive Citrobacter freundii complex and Morganella morganii (Table 1). It was interesting that these KPC-type CPE were detected in a single patient. We hypothesized that interstrain transmission of the plasmid carrying *bla*_KPC_ occurred via a mechanism involving a mobile genetic element in the patient. In the present study, we conducted whole-genome sequencing (WGS) analysis and transmission experiments with Tn*4401*-carrying plasmids to elucidate the relationship between the *bla*_KPC-2_-positive K. pneumoniae, C. freundii complex, and M. morganii strains isolated from a single patient.

**TABLE 1:** Ceftazidime-resistant CPE isolated from the single patient in this study

Strain ID	Species	Specimen	*bla*_KPC-2_	Isolation date (YYYY.MM.DD)
TUM12126	K. pneumoniae	Bile	+	201X.11.8
TUM12127	K. pneumoniae	Bile	+	201X.11.8
TUM12128	K. pneumoniae	Stool	+	201X.11.15
TUM12129	K. pneumoniae	Bile	+	201X.11.22
TUM12130	K. pneumoniae	Bile	+	201X.11.22
TUM12131	K. pneumoniae	Stool	+	201X.12.15
TUM12132	K. pneumoniae	Bile	+	201X.12.22
TUM12133	K. pneumoniae	Bile	+	201X.12.22
TUM12134	K. pneumoniae	Bile	+	201X+1.1.1
TUM12135	K. pneumoniae	Bile	+	201X+1.1.1
TUM12136	K. pneumoniae	Bile	+	201X+1.1.1
TUM12137	K. pneumoniae	Bile	+	201X+1.1.8
TUM12138	K. pneumoniae	Bile	+	201X+1.1.8
TUM12139	K. pneumoniae	Blood	+	201X+1.1.8
TUM12140	K. pneumoniae	Blood	+	201X+1.1.8
TUM12141	*C. europaeus*	Stool	−	201X.12.15
TUM12142	*C. europaeus*	Bile	−	201X.12.22
TUM12143	*C. europaeus*	Bile	−	201X.12.22
TUM12144	*C. europaeus*	Bile	−	201X+1.1.1
TUM12145	*C. europaeus*	Bile	−	201X+1.1.1
TUM12146	*C. europaeus*	Bile	−	201X+1.1.8
TUM12147	*C. europaeus*	Blood	+	201X+1.1.8
TUM12148	*C. europaeus*	Blood	+	201X+1.1.8
TUM12149	M. morganii	Bile	+	201X+1.1.1
TUM12150	M. morganii	Bile	+	201X+1.1.8
TUM12151	M. morganii	Blood	+	201X+1.1.15

## RESULTS

### Antimicrobial susceptibility and carbapenemase genes.

Twenty-six ceftazidime-resistant strains (15 K. pneumoniae, eight C. freundii complex, and three M. morganii strains) were isolated from a single patient in a university hospital in Tokyo over 3 months from November 201X to January 201X+1 (Table 1) (for the protection of personal privacy, permission was not obtained from any of the ethics committees to specify the year of isolation in the paper). The patient had been previously hospitalized in New York and Montreal before returning to Japan. All 26 strains were resistant to tazobactam-piperacillin and ceftazidime and were susceptible to amikacin (see Table S1 in the supplemental material). All 15 K. pneumoniae and all three M. morganii strains were imipenem or meropenem resistant and were determined by PCR to be *bla*_KPC_ positive. Additionally, two of the eight C. freundii complex strains with reduced susceptibility to imipenem were determined by PCR to be *bla*_KPC_ positive.

10.1128/mSphere.00850-21.4 TABLE S1 Characteristics of ceftazidime-resistant *Enterobacterales*. TAZ/PIPC, tazobactam-piperacillin; CAZ, ceftazidime; IPM, imipenem; MEPM, meropenem; ETPM, ertapenem; AZT, aztreonam; AMK, amikacin; GM, gentamicin; CPFX, ciprofloxacin. ^a^See Table S3 for Tn*4401a*-carrying plasmid information. Download Table S1, PDF file, 0.03 MB. Copyright © 2021 Sugita et al. 2021 Sugita et al. https://creativecommons.org/licenses/by/4.0/ This content is distributed under the terms of the Creative Commons Attribution 4.0 International license.

### PFGE.

All of the strains within each species, including the 15 K. pneumoniae, eight C. freundii complex (regardless of *bla*_KPC_-positive or -negative status), and three M. morganii strains, were derived from the same clone, as determined by pulsed-field gel electrophoresis (PFGE) (Fig. S1A to C).

10.1128/mSphere.00850-21.1 FIG S1 Pulsed-field gel electrophoresis (PFGE) band patterns. (A) K. pneumoniae; (B) C. freundii complex; (C) M. morganii. Download FIG S1, TIF file, 0.5 MB. Copyright © 2021 Sugita et al. 2021 Sugita et al. https://creativecommons.org/licenses/by/4.0/ This content is distributed under the terms of the Creative Commons Attribution 4.0 International license.

### Draft and complete WGS.

Draft genome sequences were obtained using an Illumina sequencer, MiSeq, for 20 *bl*a*_K_*_PC_-positive strains with an average depth of 86.6 (standard deviation [SD], 40.3) (Table S2). Assembled genomes contained an average of 78.7 (SD, 18.9) contigs and an *N*_50_ value of 176,169 bp (SD, 90,246 bp). Complete genome sequences were obtained using a Nanopore sequencer, MinION, for 15 *bl*a*_K_*_PC_-positive K. pneumoniae strains with an average depth of 332.0 (SD, 189.3) (Table S2).

10.1128/mSphere.00850-21.5 TABLE S2 Statistics of draft whole-genome sequencing data for carbapenemase-producing *Enterobacterales*. ^a^Strains sequenced by MiSeq and MinION were shown with chromosome and plasmid. Download Table S2, PDF file, 0.02 MB. Copyright © 2021 Sugita et al. 2021 Sugita et al. https://creativecommons.org/licenses/by/4.0/ This content is distributed under the terms of the Creative Commons Attribution 4.0 International license.

### Species identification and MLST.

The strains identified as K. pneumoniae, C. freundii complex, and M. morganii in the primary biochemical identification were identified as K. pneumoniae, Citrobacter europaeus, and M. morganii, respectively, by their average nucleotide identity (ANI) scores. All of the K. pneumoniae strains belonged to ST258. All of the *C. europaeus* strains were ascribed to ST497, which was a newly discovered ST in this study. No multilocus sequence typing (MLST) scheme for M. morganii is currently available.

### Antimicrobial resistance genes.

All of the *bla*_KPC_-positive strains harbored *bla*_KPC-2_, located on Tn*4401a*. The K. pneumoniae strains also possessed *bla*_OXA-9_, *bl*a_TEM-1_, *aac(3′)-IV*, *aph(4)-Ia*, *aadA1*, *aadA2*, *aac(6′)-Ib-cr*, and *cmlA1* (Table S1). The *C. europaeus* and M. morganii strains possessed *bla*_KPC-2_ and *aac(6′)-Ib-cr* (Tables S1 and S3).

10.1128/mSphere.00850-21.6 TABLE S3 Characteristics of Tn*4401a*-carrying plasmids. ^a^*bla*_KPC-2_ was harbored in Tn*4401a*. Download Table S3, PDF file, 0.02 MB. Copyright © 2021 Sugita et al. 2021 Sugita et al. https://creativecommons.org/licenses/by/4.0/ This content is distributed under the terms of the Creative Commons Attribution 4.0 International license.

### Mobile genetic element and plasmid similarity.

All 15 of the K. pneumoniae strains shared similarly structured IncN- and IncR-type multireplicon (hereafter called IncN+R) plasmids that carried Tn*4401a* (Fig. S2A and Table S3). These 15 IncN+R plasmids showed 97% to 100% coverage and 99.90% to 100% similarity to each other. In three of these K. pneumoniae strains (TUM12137, TUM12139, and TUM12140), two copies of Tn*4401a* were present on each IncN+R plasmid. No plasmids resembling the Tn*4401a*-carrying IncN+R multireplicon plasmid harbored by K. pneumoniae, represented by pMTY12126_IncN+R from TUM12126, were reported in the GenBank database prior to September 2021. The highest coverage for pMTY12126_IncN+R was 59% for K. pneumoniae BWHC1 plasmid unnamed2 (GenBank accession number CP020500.1). The IncN and IncR portions of the multireplicon pMTY12126_IncN+R were similar to plasmid 9 (IncN, GenBank accession number FJ223607.1) and pKPC484 (IncR, GenBank accession number CP008798.1), respectively (Fig. S2B). Plasmid 9 and pKPC484 showed 52% and 55% coverage of the length of pMTY12126_IncN+R, respectively (Fig. S2B). Interestingly, plasmid 9 and pKPC484 each possessed a copy of Tn*4401*, and pMTY12126_IncN+R was structured such that their copies of Tn*4401a* merged (Fig. S2B).

10.1128/mSphere.00850-21.2 FIG S2 (A) Structural comparison of the Tn*4401a*-carrying IncN+IncR plasmids in K. pneumoniae. Block arrows indicate confirmed or putative open reading frames (ORFs) and their orientations. Arrow size is proportional to the predicted ORF length. Red box indicates Tn*4401a*. The meanings of the arrow colors are as follows: green, replication initiation protein genes; blue, transposase genes; red, antibiotic resistance genes; cyan, *tra* locus genes; yellow, heavy metal resistance genes. Putative, hypothetical, and unknown genes are represented by gray arrows. (B) Structural comparison between the pMTY12126_IncR+N plasmid sequenced in this study and similar plasmids deposited in the GenBank database. Block arrows indicate confirmed or putative open reading frames (ORFs) and their orientations. Arrow size is proportional to the predicted ORF length. Red box indicates Tn*4401*. pMTY12126_IncN+R and pKPC484 were carrying Tn*4401a*, whereas plasmid 9 was carrying Tn*4401b*. The meanings of the arrow colors are as follows: green, replication initiation protein genes; blue, transposase genes; red, antibiotic resistance genes; cyan, conjugal transfer genes; yellow, heavy metal resistance genes. Putative, hypothetical, and unknown genes are represented by gray arrows. (C to E) Structural comparison of Tn*4401a*-carrying ColRNAI plasmids in K. pneumoniae and *C*. *europaeus* (C), in K. pneumoniae and M. morganii (D), or in K. pneumoniae*, C. europaeus*, and M. morganii (E). Block arrows indicate confirmed or putative open reading frames (ORFs) and their orientations. Arrow size is proportional to the predicted ORF length. Red box indicates Tn*4401a*. The meanings of the arrow colors are as follows: blue, transposase genes; red, antibiotic resistance genes; magenta, mobilization genes. Putative, hypothetical, and unknown genes are represented by gray arrows. (F) Structural comparison between the ColRNAI plasmid sequenced in this study and similar plasmids deposited in the GenBank database. Block arrows indicate confirmed or putative open reading frames (ORFs) and their orientations. Arrow size is proportional to the predicted ORF length. Red box indicates Tn*4401a*. Only regions similar to the ColRNAI plasmid are colored; except for the ORFs for replication initiation protein genes, the ORFs of other regions are shown in white. The meanings of the arrow colors are as follows: green, replication initiation protein genes; blue, transposase genes; red, antibiotic resistance genes; magenta, mobilization genes. Putative, hypothetical, and unknown genes are represented by gray arrows. Most of the ORFs in regions dissimilar to ColRNAI are indicated by white arrows. Download FIG S2, TIF file, 1.4 MB. Copyright © 2021 Sugita et al. 2021 Sugita et al. https://creativecommons.org/licenses/by/4.0/ This content is distributed under the terms of the Creative Commons Attribution 4.0 International license.

In addition, three strains of K. pneumoniae (TUM12128, TUM12134, and TUM12135) harbored Tn*4401a*-carrying ColRNAI plasmids. The Tn*4401a*-carrying ColRNAI plasmid of K. pneumoniae TUM12128 (pMTY12128_ColRNAI) had a structure identical to that of the ColRNAI plasmids of *C. europaeus* TUM12147 (pMTY12147_ColRNAI) and TUM12148 (pMTY12148_ColRNAI) (Fig. S2C). Six *bla*_KPC_-negative *C. europaeus* strains (from TUM12141 to TUM12146) were ColRNAI plasmid-specific PCR (*mobB*) negative.

Similarly, the Tn*4401a*-carrying ColRNAI plasmids of TUM12134 (pMTY12134_ColRNAI) and TUM12135 (pMTY12135_ColRNAI) had structures identical to those of the ColRNAI plasmids of M. morganii TUM12149, TUM12150, and TUM12151 (pMTY12149_ColRNAI, pMTY12150_ColRNAI, and pMTY12151_ColRNAI, respectively) (Fig. S2D). The backbone structures of the Tn*4401a*-carrying ColRNAI plasmids in this study were all identical, the only difference being the Tn*4401a* insertion site (Fig. S2E). The structures of the Tn*4401a*-carrying ColRNAI plasmids, pMTY12147_ColRNAI and pMTY12150_ColRNAI, were similar only to that of plasmid 15S (GenBank accession number FJ223606.1) among the identified plasmids, differing only in the position in which Tn*4401a* was inserted (Fig. S2F). The coverage of pMTY12147_ColRNAI, pMTY12150_ColRNAI, and plasmid 15S was 100%, with the only difference being the insertion site of Tn*4401a*. In addition, plasmids with structures that appear to be integrations of the Tn*4401*a-carrying ColRNAI plasmid into the IncFIB/FII, IncFII, or IncR plasmid are registered in the GenBank database (Fig. S2F).

### Core-genome-based SNP analysis of K. pneumoniae strains and their Tn*4401a*-carrying plasmids.

**FIG 1:**
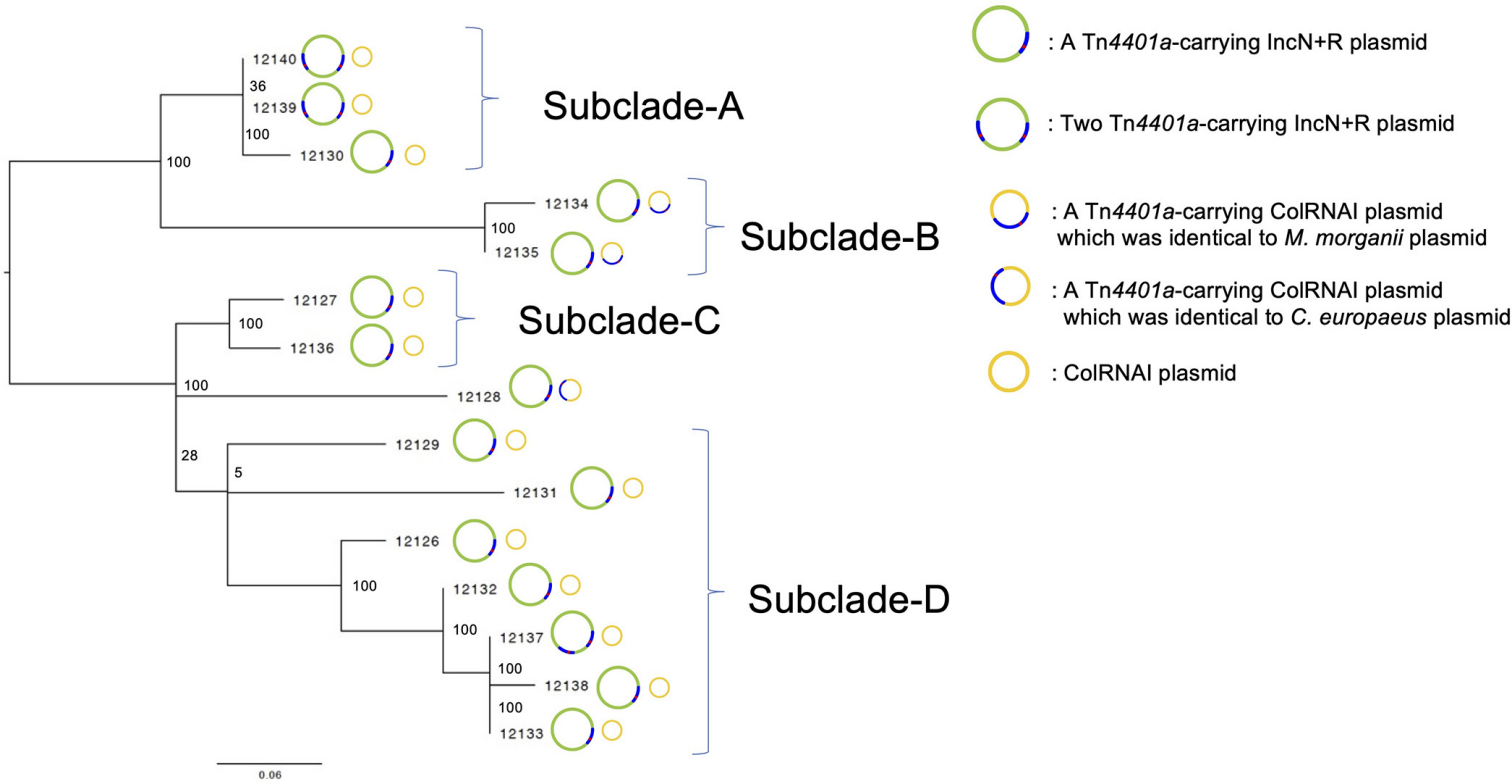
Phylogenetic tree of *bla*_KPC-2_-positive Klebsiella pneumoniae strains. Phylogenetic tree of the 15 *bla*_KPC-2_-positive K. pneumoniae strains and information on their harbored plasmids. The phylogenetic tree was constructed using a maximum-likelihood phylogenetic analysis based on single nucleotide polymorphisms (SNPs) in the core genome and excluding homologous recombination sequences. We used the bootstrap 1,000 times and listed the bootstrap values on the branch. The core-genome region comprised 95.1% (5,130,070/5,394,056 bp) of the genome of the reference strain, K. pneumoniae KPNIH1 ST258 (GenBank accession number NZ_CP008827.1). The scale distance corresponds to the number of SNPs per the core genome, excluding the homologous recombination sequence.

Phylogenetic analysis of the 15 K. pneumoniae strains from this study and 20 unrelated *bla*_KPC-2_-positive ST258 K. pneumoniae strains isolated overseas for which genomic data are available from the GenBank database (Table S4) revealed that the 15 strains from this study form a cluster (Fig. S3A). The average substitution rate in the core genome was estimated to be 1.32 single nucleotide polymorphisms (SNPs) (95% highest posterior density interval, 0.19 to 2.46 SNPs) per genome per year, and the time of divergence of the 15-strain cluster in this study from the other analyzed strains was estimated to be approximately 100 months (approximately 8 years) before the time when these strains were isolated in a Japanese hospital (Fig. S3A). Phylogenetic analysis of only the 15 K. pneumoniae strains from this study showed that these strains can be divided into four subclades (subclades A, B, C, and D) (Fig. 1). The maximum number of SNPs among all 15 strains was 18, and the maximum number of SNPs among each subclade was only two (Fig. S3B). Two of the three strains in subclade A and one of the seven strains in subclade D harbored two copies of Tn*4401a* on their IncN+R plasmid (Fig. 1). All strains in subclade B (TUM12134 and TUM12135) and one strain not belonging to a subclade (TUM12128) harbored a ColRNAI plasmid carrying Tn*4401a* (Fig. 1).

10.1128/mSphere.00850-21.3 FIG S3 (A) A time-calibrated phylogenetic tree of *bla*_KPC-2_-positive K. pneumoniae strains. Phylogenetic tree, calibrated by time in months, of the 15 *bla*_KPC-2_-positive K. pneumoniae strains from the present study and 20 unrelated *bla*_KPC-2_-positive K. pneumoniae ST258 strains whose sequences were downloaded from the GenBank database (Table S4). Using BEAST (version 2.4.7), the phylogenetic tree was constructed based on single nucleotide polymorphisms (SNPs) in the core genome and excluding homologous recombination sequences. The core-genome region comprised 83.7% (4,515,415/5,394,056 bp) of the genome of the reference strain, K. pneumoniae KPNIH1 ST258 (GenBank accession number NZ_CP008827.1). The scale distance corresponds to the number of SNPs per the core genome, excluding the homologous recombination sequence. The 95% highest posterior density (HPD) at the time of phylogenetic divergence is indicated by purple bars. (B) Matrix representation of the number of SNPs on the core genome in *bla*_KPC-2_-positive K. pneumoniae. The notation order matches that of Fig. 1. The prefix TUM was omitted. Download FIG S3, TIF file, 1.1 MB. Copyright © 2021 Sugita et al. 2021 Sugita et al. https://creativecommons.org/licenses/by/4.0/ This content is distributed under the terms of the Creative Commons Attribution 4.0 International license.

10.1128/mSphere.00850-21.7 TABLE S4 GenBank database information of 20 unrelated *bla*_KPC-2_-positive ST258 K. pneumoniae strains. NA, not available. Download Table S4, PDF file, 0.01 MB. Copyright © 2021 Sugita et al. 2021 Sugita et al. https://creativecommons.org/licenses/by/4.0/ This content is distributed under the terms of the Creative Commons Attribution 4.0 International license.

### Plasmid transfer and similarity.

The Tn*4401a*-carrying IncN+R plasmids from all except one of the K. pneumoniae strains (pMTY12128_IncN+R from TUM12128) were successfully transferred to Escherichia coli by conjugation. Interestingly, the pMTY12128_IncN+R plasmid, which was unable to be transferred by conjugation, had an approximately 20-kb inversion in the region of the conjugative transfer-associated gene cluster (Fig. S2A).

MOB-suite software detected the *mobC* gene in all ColRNAI plasmids with or without Tn*4401a*, which could not be detected by DFAST, and determined it to be a mobilizable plasmid. Conjugative transfer experiments revealed that the pMTY12133_ColRNAI and pMTY12134_ColRNAI plasmids could be successfully mobilized from K. pneumoniae TUM12133 and TUM12134, respectively, into E. coli via the conjugative transfer of an IncN+R plasmid. Plasmids pMTY12148_ColRNAI and pMTY12151_ColRNAI were also successfully mobilized by transforming the ColRNAI plasmid in E. coli harboring a self-transferable IncW plasmid, as we previously reported (*bla*_IMP-1_-carrying plasmid, pMTY10660_IncW, GenBank accession number AP018350.1) (26). The E. coli harboring a *bla*_IMP-1_-carrying IncW plasmid and a *bla*_KPC-2_-carrying ColRNAI plasmid showed resistance to both moxalactam (>4 mg/liter) and aztreonam (>4 mg/liter) on selective agar plates.

## DISCUSSION

In this study, we estimated the transposition of Tn*4401a* from an IncN+R plasmid to a ColRNAI plasmid in K. pneumoniae strains and found that the Tn*4401a*-carrying ColRNAI plasmid mobilized via conjugal plasmid transfer to the recipient. Furthermore, the mobilization of Tn*4401a*-carrying ColRNAI plasmids was confirmed by the results of *in vitro* conjugation experiments using E. coli as the recipient.

The Tn*4401a*-carrying IncN+R and ColRNAI plasmids that we analyzed were both found to be novel structures with low similarity to plasmids currently registered in the GenBank database. The IncR plasmid has been reported to form a multireplicon with the IncA/C and IncN plasmids (27). To the best of our knowledge, no IncR plasmid with conjugative ability has been reported, and we speculate that the IncN+R plasmid described in this study acquired conjugative ability by forming a multireplicon, as previously reported (28). The Tn*4401a*-carrying IncN+R plasmid may have acquired conjugative transfer ability by inheriting the *tra* gene from an ancestral IncN plasmid similar to plasmid 9.

The results of a core-genome SNP-based phylogenetic analysis conducted on 15 *bla*_KPC-2_-positive K. pneumoniae strains from this study and representative ST258 strains from the GenBank database suggested that the strains from our patient diverged and evolved independently approximately 8 years prior to their time of isolation (see Fig. S3A in the supplemental material). This result strongly suggests that the patient may have already been carrying *bla*_KPC-2_-positive ST258 K. pneumoniae strains at the time of medical treatment in New York, USA, and/or Montreal, Canada. Tn*4401a* detected in this study has the same structure as the IncFII-like plasmids pKpQIL (GenBank accession number GU595196.1) and pNYC (GenBank accession number EU176011.1) (21). Tn*4401* is a transposon belonging to the Tn*3* family and has been reported to be actively translocated in bacteria (29).

The WGS analysis of our 15 K. pneumoniae strains revealed that Tn*4401a* replicated on the IncN+R plasmid, leading to the generation over a 3-month period of bacteria harboring two copies, which it replicatively transferred to ColRNAI plasmids (Fig. S2A). Because ColRNAI plasmids carrying *bla*_KPC-2_ were found to be incapable of conjugative transduction themselves due to the lack of conjugative transfer element (*tra*) genes (Fig. S2C), it is likely that the conjugative transduction ability of the IncN+R plasmid helped K. pneumoniae to transmit Tn*4401a* to *C. europaeus* and M. morganii. However, the IncN+R plasmid was not detected in *C. europaeus* or M. morganii, suggesting that the plasmid was not adapted to these hosts. Transposition of Tn*4401a* to ColRNAI must have been essential for the strains of *C. europaeus* and M. morganii in this patient to acquire *bla*_KPC-2_. From the WGS data, we speculate that Tn*4401a* on the ColRNAI plasmids may have been sourced from an IncN+R plasmid in K. pneumoniae; however, to date, we have not experimentally demonstrated that Tn*4401* was transferred.

This study revealed novel IncN+R and ColRNAI plasmids carrying Tn*4401a*. The data suggest that the interplasmid transposition of Tn*4401a* from an IncN+R plasmid to a ColRNAI plasmid in K. pneumoniae and the mobilization of Tn*4401a*-carrying ColRNAI plasmids contributed to the acquisition of *bla*_KPC-2_ in strains of *C. europaeus* and M. morganii.

## MATERIALS AND METHODS

### Bacterial strains.

The bacterial strains used in the present study were isolated from a single patient in a university hospital in Tokyo over the 3-month period from November 201X to January 201X+1. The strains comprised 26 ceftazidime-resistant strains (15 K. pneumoniae, eight C. freundii complex, and three M. morganii) isolated from a patient who was hospitalized in New York and Montreal before returning to Japan. The strains were isolated during routine testing of bile and blood samples using sheep blood agar (Nissui Pharmaceutical Co., Ltd., Tokyo, Japan) and MacConkey II agar (Becton, Dickinson, Franklin Lakes, NJ, USA), and during active surveillance of extended-spectrum cephalosporins or carbapenem-resistant *Enterobacterales* in stool samples using ChromID ESBL agar (bioMérieux, Lyon, France). The patient had received multiple antibiotics, including meropenem, during the previous month.

### Ethics.

The study protocol was approved by the Ethics Committee of the Toho University School of Medicine (no. 27037 and A17023) and the Keio University School of Medicine (no. 20150090).

### Primary bacterial species identification and antimicrobial susceptibility testing.

Primary bacterial species identification and antibiotic susceptibility testing were performed with NBPcomb6.23J and Neg MIC6.31J of MicroScan WalkAway Plus (Beckman Coulter, Brea, CA, USA). Strains showing MICs of 4 mg/liter or higher for ceftazidime were judged as potential carbapenemase producers. The MICs were measured using the broth microdilution method in accordance with the CLSI guidelines (M07, 11th edition) (30). The following antimicrobial agents were used for antimicrobial susceptibility testing: piperacillin, ceftazidime, and imipenem (Sigma Chemical, St. Louis, MO, USA); ciprofloxacin (LKT Laboratories, St Paul, MN, USA); aztreonam (Tokyo Chemical Industry Co. Ltd., Tokyo, Japan); tazobactam (Toyama Chemical Co., Ltd., Toyama, Japan); meropenem, amikacin, and gentamicin (Wako Pure Chemical Industries, Ltd., Tokyo, Japan); and ertapenem (Merck & Co., Inc., Kenilworth, NJ, USA). The MIC measurement range was 0.125 to 256 mg/liter. E. coli ATCC 25922 and Pseudomonas aeruginosa ATCC 27853 were used as quality control strains for antibiotic susceptibility testing. The results were interpreted in accordance with the CLSI guidelines (M100, 31st edition) (31).

### Screening carbapenemase genes by PCR.

The main carbapenemase genes, *bla*_KPC_, *bla*_NDM_, *bla*_IMP_, *bla*_VIM_, and *bl*a_OXA-48-like_, were screened using conventional PCR (32). Carbapenemase gene-positive strains were used in the following analyses in this study.

### PFGE.

K. pneumoniae, C. freundii complex, and M. morganii genomic DNAs, in gel-embedded form, were digested with XbaI (TaKaRa Bio, Shiga, Japan). Pulsed-field gel electrophoresis (PFGE) was performed using CHEF Mapper (Bio-Rad, Hercules, CA, USA) in 1× Tris-borate-EDTA (TBE) buffer, along with Lambda Ladder PFG marker (New England Biolabs, Hertfordshire, United Kingdom). Fingerprinting II software (Bio-Rad) was used to analyze the electrophoretic patterns, and the analysis parameters were as follows: the Dice coefficient, the unweighted pair group method with averages, 1% position tolerance, and 1% optimization.

### Draft WGS and analysis.

DNA was extracted from bacterial cultures, grown in LB medium using phenol-chloroform, and was purified using a QIAamp PCR purification kit (Qiagen, Valencia, CA, USA). DNA libraries were created using the Nextera XT DNA sample preparation kit (Illumina, Inc., San Diego, CA, USA). The DNA libraries were sequenced with the MiSeq platform (Illumina) for paired-end reads of 300 bp using a MiSeq reagent kit v3 600-cycle kit. MiSeq output reads were assembled with SPAdes v3.13.1 (33). Species identification was performed using the ANI with the type strain database gcType (34) and the comparesketch command in the BBMap package (https://sourceforge.net/projects/bbmap/) (35). We used a cutoff value of ≥96% (36) of the ANI value compared with the genomic sequences of the type strain for species identification. The DNA Data Bank of Japan Fast Annotation and Submission Tool (DFAST) was used for open reading frame (ORF) and gene annotation (37). ResFinder and Plasmid Finder from the Center for Genomic Epidemiology (https://cge.cbs.dtu.dk/services/) were used to detect acquired antimicrobial resistance genes and plasmid replicons. Multilocus sequence typing (MLST) was performed using the PubMLST scheme for K. pneumoniae and *Citrobacter* spp. (https://pubmlst.org/). *In silico* relaxase typing (MOB typing) was performed with MOB-suite (38).

### MinION sequencing and hybrid *de novo* assembly with MiSeq reads.

To determine the complete sequences of *bla*_KPC-2_-carrying plasmids, genomic DNA was sequenced using MinION (Oxford Nanopore Technologies, Oxford, United Kingdom). DNA extraction and library preparation were performed using a NucleoBond AXG 20 column (TaKaRa Bio) combined with NucleoBond buffer set III (TaKaRa Bio) and rapid barcoding kit SQK-RBK004 (Oxford Nanopore Technologies), respectively. MinION flow cell R9.4 (Oxford Nanopore Technologies) was used for sequencing. Basecalling and demultiplexing were performed by Guppy v3.4.1. Hybrid *de novo* assembly, using both MiSeq and MinION reads, was conducted with Unicycler (39) after the reads were quality trimmed with the Trimmomatic tool (version 0.38) (40) and NanoFilt (41).

### Core-genome SNP-based phylogenetic analysis.

Core-genome single nucleotide polymorphism (SNP)-based phylogenetic analysis was performed with MiSeq sequencing data. The MiSeq sequencing data were aligned to the genomic sequence of the reference isolate, K. pneumoniae KPNIH1 ST258 (GenBank accession number NZ_CP008827.1), using the Burrows-Wheeler Aligner (BWA) with the “SW” option (42). We constructed a core-genome alignment using SAMtools (version 1.3) mpileup (43), and VarScan (version 2.3.7) mpileup2cns (44) and then a maximum-likelihood tree using PhyML (45). Using this as the starting tree, we inferred homologous recombination events that imported DNA fragments from beyond the phylogenetic clade and constructed a clonal phylogeny with corrected branch lengths using ClonalFrameML (46). The core genome, excluding homologous recombination sequences estimated using ClonalFrameML, was subjected to SNP detection and used for phylogenetic analysis by RAxML (47). We used the bootstrap 1,000 times and the gamma site model (GTR) substitution model. In addition, we estimated the evolutionary rate and timing of phylogenetic divergence of the 15 K. pneumoniae strains isolated over 3 months by BEAST (version 2.4.7) (48). BEAST was run for 20 million generations, sampling every 200 states, using the GTR substitution model.

### Conjugation experiments.

The conjugation experiments were performed by applying the filter mating method. A sodium azide- and rifampin-resistant, lactose-nonfermenting E. coli strain (ML4909 with added sodium azide resistance) was used as the recipient (26). Transconjugants were selected on modified Drigalski agar (Eiken Chemical Co., Ltd., Tokyo, Japan) containing both ceftazidime (4 mg/liter) and sodium azide (100 mg/liter; Fujifilm Wako Pure Chemical Corporation, Osaka, Japan). The carriage of *bla*_KPC-2_ by transconjugants was confirmed by PCR (32). Confirmation of the transmitted plasmid replicon type was performed by IncN- and IncR-specific PCR (23, 49).

### Mobilization experiments.

The procedure applied for confirming that the horizontal transfer to recipient cells occurred by the mobilization modality of a ColRNAI plasmid carrying *bla*_KPC-2_, which did not appear to be self-transmissive by conjugation, was as follows. First, the test plasmids (*bla*_KPC-2_-carrying ColRNAI plasmids designated pMTY12148_ColRNAI and pMTY12151_ColRNAI) were introduced by electroporation into E. coli cells carrying a conjugative *bla*_IMP-1_-carrying plasmid (pMTY10660_IncW). The E. coli cells were washed three times with ice-cooled sterilized water as a pretreatment for electroporation. The E. coli Pulser (Bio-Rad, Munich, Germany) was used at a voltage of 1.5 kV. Second, a conjugation experiment was performed using an E. coli strain harboring a *bla*_IMP-1_-carrying IncW plasmid and a *bla*_KPC-2_-carrying ColRNAI plasmid as the donor and rifampin-resistant E. coli ML4909 as the recipient. If the *bla*_IMP-1_- and *bla*_KPC-2_-carrying plasmids were transferred at the same time, mobilization was considered to have occurred. Therefore, the *bla*_KPC-2_-carrying mobilized cells were selected by their growth on modified Drigalski agar containing the three antibiotics aztreonam (4 mg/liter), moxalactam (4 mg/liter; Shionogi & Co., Ltd., Osaka, Japan), and rifampin (50 mg/liter; Fujifilm Wako Pure Chemical Corporation, Osaka, Japan). Confirmation of the transmitted *bla*_IMP-1_ and *bla*_KPC-2_ genes (32) and ColRNAI plasmid was performed by PCR. Transfer of the ColRNAI plasmid was confirmed by PCR of *mobB*, which is specifically retained by the ColRNAI plasmid. The primers used for this amplification were *mobB*-Fw (5′-ATCTGTTCCGCGATCTCGAC-3′) and *mobB*-Rv (5′-CCCCAGTGCGTTCAGTACAT-3′).

### Accession number(s).

The draft and whole-genome sequencing data were deposited under the GenBank BioProject accession number PRJDB11646. The accession numbers for each strain and plasmid are shown in Tables S1 and S3 in the supplemental material.
