# Supplementary material for: Molecular Analysis of blaKPC-2-Harboring Plasmids: Tn4401a Interplasmid Transposition and Tn4401a-Carrying ColRNAI Plasmid Mobilization from Klebsiella pneumoniae to Citrobacter europaeus and Morganella morganii in a Single Patient
Source: mSphere. 2021 Nov 3;6(6):e00850-21. doi: 10.1128/mSphere.00850-21 (PMC8565517; doi:10.1128/mSphere.00850-21)
Supplement: TABLE S1 [file msphere.00850-21-st001.pdf]

			MIC (mg/L)													
Species by ANI	Strain ID	Collection date (YYYY.MM.DD)	TAZ/PIPC	CAZ	IPM	MEPM	ETPM	AZT	AMK	GM	CPFX	KPC type	MLST	Aquired antimicrobial resistance genes	Harbored plamsid replicon type[a]	Accession ID
	TUM12126	201x.11.09	>256/4	256	16	32	128	>256	16	8	256	KPC-2	ST258	*bla*$_{KPC-2}$, *bla*$_{OXA-9}$, *bla*$_{TEM-1}$, *aph(4)-Ia, aac(6')-Ib-cr, aadA1, aac(3)-IV, aadA2, cmlA1*	IncN+R, ColRNAI	AP024750-AP024752
	TUM12127	201x.11.09	>256/4	256	16	16	64	>256	16	16	64	KPC-2	ST258	*bla*$_{KPC-2}$, *bla*$_{OXA-9}$, *bla*$_{TEM-1}$, *aph(4)-Ia, aac(6')-Ib-cr, aadA1, aac(3)-IV, aadA2, cmlA1*	IncN+R, ColRNAI	AP024753-AP024755
	TUM12128	201x.11.18	>256/4	256	32	64	256	>256	16	16	128	KPC-2	ST258	*bla*$_{KPC-2}$, *bla*$_{OXA-9}$, *bla*$_{TEM-1}$, *aph(4)-Ia, aac(6')-Ib-cr, aadA1, aac(3)-IV, aadA2, cmlA1*	IncN+R, ColRNAI	AP024756-AP024759
	TUM12129	201x.11.23	>256/4	>256	16	32	128	>256	16	32	256	KPC-2	ST258	*bla*$_{KPC-2}$, *bla*$_{OXA-9}$, *bla*$_{TEM-1}$, *aph(4)-Ia, aac(6')-Ib-cr, aadA1, aac(3)-IV, aadA2, cmlA1*	IncN+R, ColRNAI	AP024760-AP024762
	TUM12130	201x.11.23	>256/4	>256	32	64	256	>256	16	8	128	KPC-2	ST258	*bla*$_{KPC-2}$, *bla*$_{OXA-9}$, *bla*$_{TEM-1}$, *aph(4)-Ia, aac(6')-Ib-cr, aadA1, aac(3)-IV, aadA2, cmlA1*	IncN+R, ColRNAI	AP024763-AP024765
	TUM12131	201x.12.20	>256/4	256	16	16	128	>256	16	16	64	KPC-2	ST258	*bla*$_{KPC-2}$, *bla*$_{OXA-9}$, *bla*$_{TEM-1}$, *aph(4)-Ia, aac(6')-Ib-cr, aadA1, aac(3)-IV, aadA2*	IncN+R, ColRNAI	AP024766-AP024768
	TUM12132	201x.12.24	>256/4	256	8	32	128	>256	16	8	128	KPC-2	ST258	*bla*$_{KPC-2}$, *bla*$_{OXA-9}$, *bla*$_{TEM-1}$, *aph(4)-Ia, aac(6')-Ib-cr, aadA1, aac(3)-IV, aadA2, cmlA1*	IncN+R, ColRNAI	AP024769-AP024771
K. pneumoniae	TUM12133	201x.12.24	>256/4	256	64	256	>256	>256	16	16	128	KPC-2	ST258	*bla*$_{KPC-2}$, *bla*$_{OXA-9}$, *bla*$_{TEM-1}$, *aph(4)-Ia, aac(6')-Ib-cr, aadA1, aac(3)-IV, aadA2, cmlA1*	IncN+R, ColRNAI	AP024772-AP024774
	TUM12134	201x+1.01.05	>256/4	>256	32	64	256	>256	16	8	64	KPC-2	ST258	*bla*$_{KPC-2}$, *bla*$_{OXA-9}$, *bla*$_{TEM-1}$, *aph(4)-Ia, aac(6')-Ib-cr, aadA1, aac(3)-IV, aadA2, cmlA1*	IncN+R, ColRNAI	AP024775-AP024777
	TUM12135	201x+1.01.05	>256/4	>256	32	64	256	>256	16	64	64	KPC-2	ST258	*bla*$_{KPC-2}$, *bla*$_{OXA-9}$, *bla*$_{TEM-1}$, *aph(4)-Ia, aac(6')-Ib-cr, aadA1, aac(3)-IV, aadA2, cmlA1*	IncN+R, ColRNAI	AP024778-AP024780
	TUM12136	201x+1.01.05	>256/4	256	16	32	256	>256	16	32	128	KPC-2	ST258	*bla*$_{KPC-2}$, *bla*$_{OXA-9}$, *bla*$_{TEM-1}$, *aph(4)-Ia, aac(6')-Ib-cr, aadA1, aac(3)-IV, aadA2, cmlA1*	IncN+R, ColRNAI	AP024781-AP024783
	TUM12137	201x+1.01.12	>256/4	>256	16	64	256	>256	8	16	128	KPC-2	ST258	*bla*$_{KPC-2}$ (two copies), *bla*$_{OXA-9}$, *bla*$_{TEM-1}$, *aph(4)-Ia, aac(6')-Ib-cr, aadA1, aac(3)-IV, aadA2, cmlA1*	IncN+R, ColRNAI, IncFIB	AP024784-AP024787
	TUM12138	201x+1.01.12	>256/4	128	64	128	>256	>256	16	16	128	KPC-2	ST258	*bla*$_{KPC-2}$, *bla*$_{OXA-9}$, *bla*$_{TEM-1}$, *aph(4)-Ia, aac(6')-Ib-cr, aadA1, aac(3)-IV, aadA2, cmlA1*	IncN+R, ColRNAI, IncFIB	AP024788-AP024791
	TUM12139	201x+1.01.12	>256/4	256	16	32	256	>256	16	16	64	KPC-2	ST258	*bla*$_{KPC-2}$ (two copies), *bla*$_{OXA-9}$, *bla*$_{TEM-1}$, *aph(4)-Ia, aac(6')-Ib-cr, aadA1, aac(3)-IV, aadA2, cmlA1*	IncN+R, ColRNAI	AP024792-AP024794
	TUM12140	201x+1.01.12	>256/4	256	16	32	256	>256	16	16	64	KPC-2	ST258	*bla*$_{KPC-2}$ (two copies), *bla*$_{OXA-9}$, *bla*$_{TEM-1}$, *aph(4)-Ia, aac(6')-Ib-cr, aadA1, aac(3)-IV, aadA2, cmlA1*	IncN+R, ColRNAI	AP024795-AP024797
	TUM12141	201x.12.20	64/4	256	0.5	≦0.125	0.25	128	1	0.25	≦0.125	Negative	NA	NA	ColRNAI specific PCR was negative	NA
	TUM12142	201x.12.24	64/4	>256	0.5	≦0.125	0.25	128	0.5	0.25	≦0.125	Negative	NA	NA	ColRNAI specific PCR was negative	NA
	TUM12143	201x.12.24	32/4	128	0.5	≦0.125	0.25	128	1	0.25	≦0.125	Negative	NA	NA	ColRNAI specific PCR was negative	NA
C. europaeus	TUM12144	201x+1.01.05	32/4	256	0.5	≦0.125	≦0.125	64	0.5	≦0.125	0.25	Negative	NA	NA	ColRNAI specific PCR was negative	NA
	TUM12145	201x+1.01.05	64/4	256	0.5	≦0.125	0.25	128	1	0.25	≦0.125	Negative	NA	NA	ColRNAI specific PCR was negative	NA
	TUM12146	201x+1.01.12	64/4	>256	0.5	≦0.125	0.25	128	1	0.25	≦0.125	Negative	NA	NA	ColRNAI specific PCR was negative	NA
	TUM12147	201x+1.01.12	>256/4	>256	1	2	4	>256	16	0.25	≦0.125	KPC-2	ST497	*bla*$_{KPC-2}$, *aac(6')-Ib-cr*	ColRNAI	BPMF01000001-BPMF01000087
	TUM12148	201x+1.01.12	64/4	>256	1	1	4	128	16	0.25	≦0.125	KPC-2	ST497	*bla*$_{KPC-2}$, *aac(6')-Ib-cr*	ColRNAI	BPMG01000001-BPMG01000034
	TUM12149	201x+1.01.05	64/4	64	8	1	0.5	32	8	1	≦0.125	KPC-2	ND	*bla*$_{KPC-2}$, *aac(6')-Ib-cr*	ColRNAI	BPMH01000001-BPMH01000122
M. morganii	TUM12150	201x+1.01.12	256/4	64	4	1	0.5	128	8	1	≦0.125	KPC-2	ND	*bla*$_{KPC-2}$, *aac(6')-Ib-cr*	ColRNAI	BPMI01000001-BPMI01000077
	TUM12151	201x+1.01.19	>256/4	32	8	1	1	4	8	1	≦0.125	KPC-2	ND	*bla*$_{KPC-2}$, *aac(6')-Ib-cr*	ColRNAI	BPMJ01000001-BPMJ01000044
