# Supplementary figures and images for: Molecular Analysis of blaKPC-2-Harboring Plasmids: Tn4401a Interplasmid Transposition and Tn4401a-Carrying ColRNAI Plasmid Mobilization from Klebsiella pneumoniae to Citrobacter europaeus and Morganella morganii in a Single Patient
Source: mSphere. 2021 Nov 3;6(6):e00850-21. doi: 10.1128/mSphere.00850-21 (PMC8565517; doi:10.1128/mSphere.00850-21)

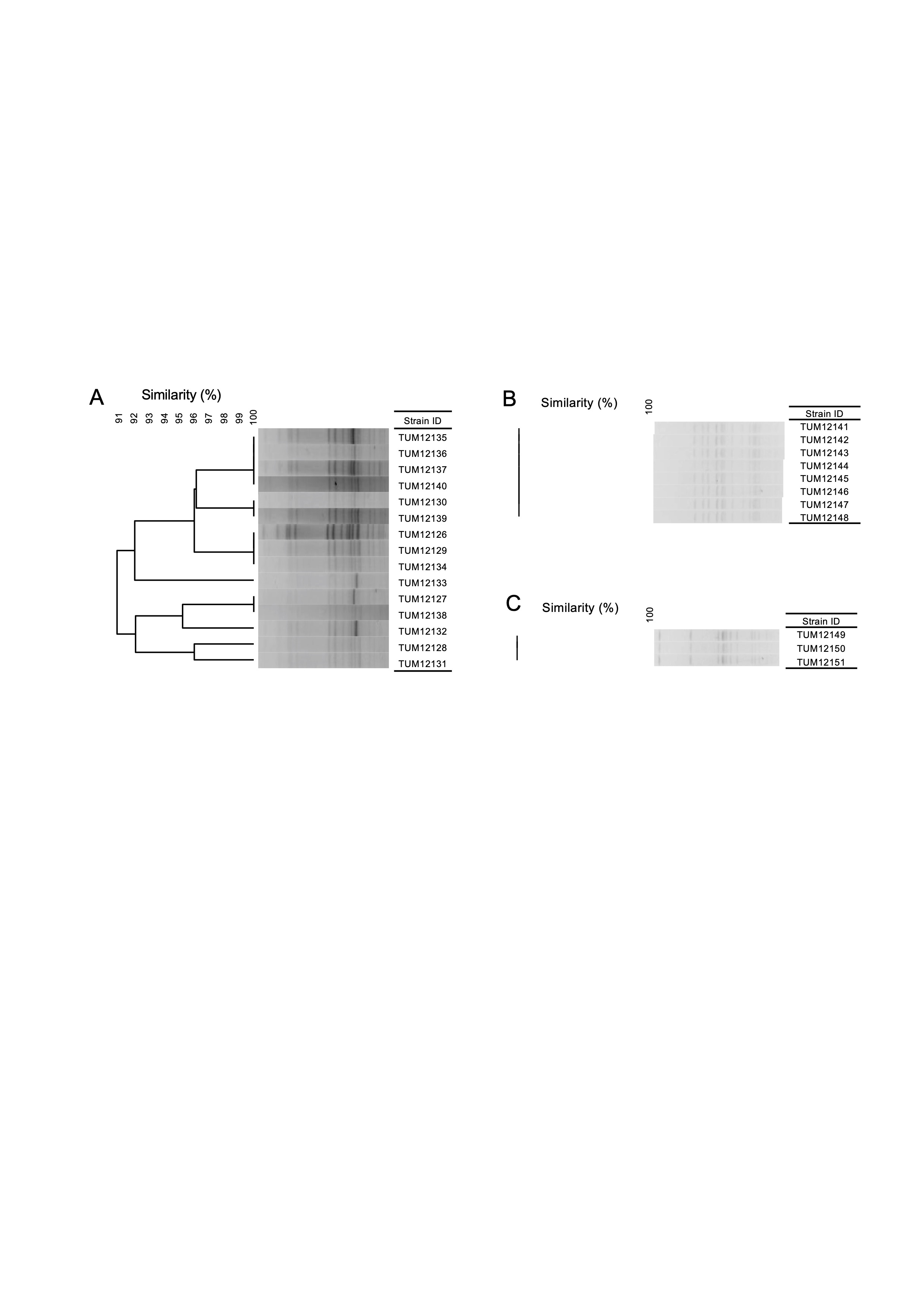

Supplement: FIG S1 [file msphere.00850-21-sf001.tif]

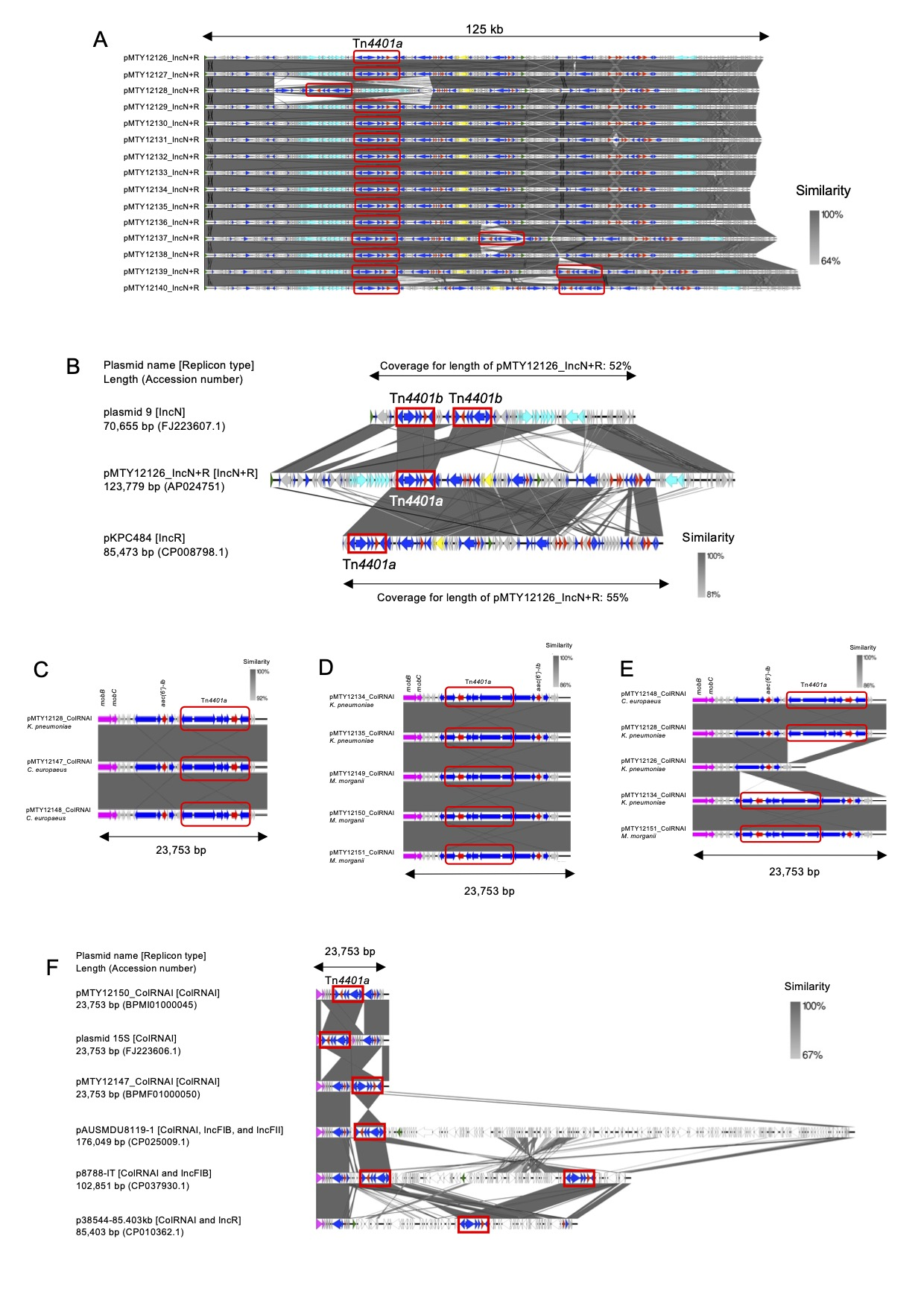

Supplement: FIG S2 [file msphere.00850-21-sf002.tif]

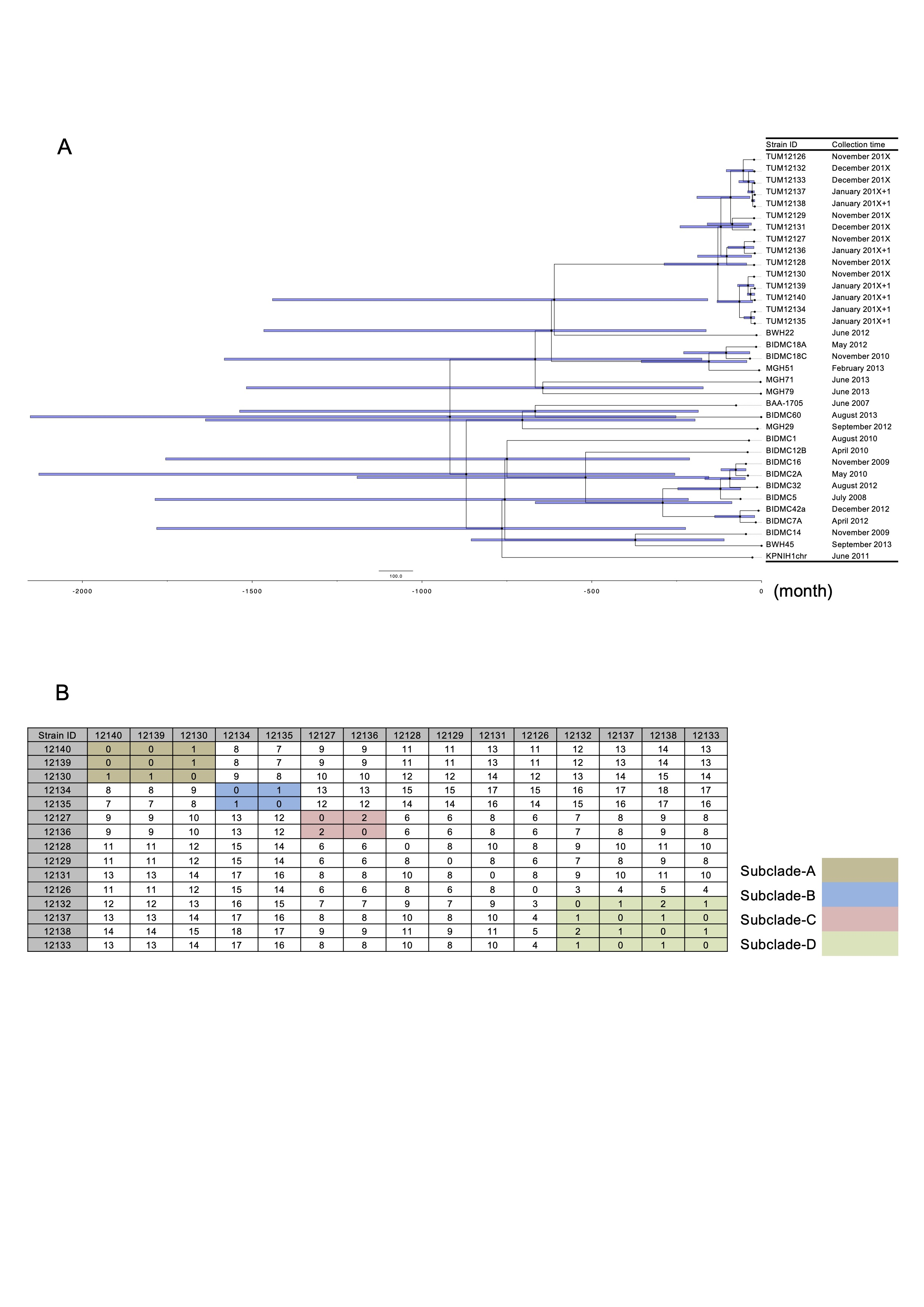

Supplement: FIG S3 [file msphere.00850-21-sf003.tif]
