# Supplementary material for: Molecular Analysis of blaKPC-2-Harboring Plasmids: Tn4401a Interplasmid Transposition and Tn4401a-Carrying ColRNAI Plasmid Mobilization from Klebsiella pneumoniae to Citrobacter europaeus and Morganella morganii in a Single Patient
Source: mSphere. 2021 Nov 3;6(6):e00850-21. doi: 10.1128/mSphere.00850-21 (PMC8565517; doi:10.1128/mSphere.00850-21)
Supplement: TABLE S2 [file msphere.00850-21-st002.pdf]

Strain ID	Sequence platform	Genome size[a]	GC (%)	MiSeq					MinION		
				No. of contigs	N50 (bp)	No. of reads	No. of nucleotide	depth (×)	No. of reads	Total >Q5 nucleotide (Mb)	depth (×)
TUM12126	MiSeq and MinION	5,429,442	57.42	71	153,594	153,594	275,257,103	38	73,574	938.9	173
TUM12127	MiSeq and MinION	5,489,659	57.35	85	157,687	157,687	397,886,632	56	80,796	1,067.5	194
TUM12128	MiSeq and MinION	5,349,608	57.36	77	164,558	164,558	1,092,122,442	147	79,344	1,460.1	273
TUM12129	MiSeq and MinION	5,491,076	57.35	82	203,931	203,931	303,548,823	43	88,908	1,266.8	231
TUM12130	MiSeq and MinION	5,474,895	57.36	75	172,948	172,948	330,663,222	48	192,510	2,368.7	433
TUM12131	MiSeq and MinION	5,430,376	57.41	73	173,152	173,152	288,077,804	41	44,395	614.8	113
TUM12132	MiSeq and MinION	5,423,150	57.44	72	169,111	169,111	421,004,728	62	106,299	1,948.5	359
TUM12133	MiSeq and MinION	5,431,127	57.43	67	169,111	169,111	353,820,337	50	69,979	1,273.4	234
TUM12134	MiSeq and MinION	5,435,736	57.42	73	168,277	168,277	980,908,162	127	129,934	2,332.9	429
TUM12135	MiSeq and MinION	5,430,008	57.43	106	136,012	136,012	887,890,831	114	102,668	1,851.9	341
TUM12136	MiSeq and MinION	5,490,704	57.35	82	159,237	159,237	319,308,833	44	182,614	3,463.3	631
TUM12137	MiSeq and MinION	5,644,338	56.97	83	159,237	159,237	1,197,559,074	144	87,559	1,068.2	189
TUM12138	MiSeq and MinION	5,639,316	56.96	98	136,081	136,081	613,967,214	80	41,464	807.1	143
TUM12139	MiSeq and MinION	5,498,736	57.35	78	198,932	198,932	388,268,432	54	229,804	4,326.3	787
TUM12140	MiSeq and MinION	5,500,734	57.35	88	159,237	159,237	1,016,668,939	113	166,515	2,473.7	450
TUM12147	MiSeq	5,125,994	52.11	87	139,152	139,152	787,401,934	123	NA	NA	NA
TUM12148	MiSeq	5,133,369	52.08	34	530,134	530,134	889,813,658	129	NA	NA	NA
TUM12149	MiSeq	3,732,426	51.25	122	53,599	53,599	368,686,832	76	NA	NA	NA
TUM12150	MiSeq	3,746,050	51.20	77	105,014	105,014	449,840,501	95	NA	NA	NA
TUM12151	MiSeq	3,750,047	51.17	44	214,367	214,367	773,508,571	150	NA	NA	NA
Average	-	-	-	78.7	176,169	176,169	606,810,204	86.6	111757.5	1817.5	332.0
SD	-	-	-	18.9	90,246	90,246	311,674,145	40.3	56516.1	1038.9	189.3
