# Supplementary material for: Molecular Analysis of blaKPC-2-Harboring Plasmids: Tn4401a Interplasmid Transposition and Tn4401a-Carrying ColRNAI Plasmid Mobilization from Klebsiella pneumoniae to Citrobacter europaeus and Morganella morganii in a Single Patient
Source: mSphere. 2021 Nov 3;6(6):e00850-21. doi: 10.1128/mSphere.00850-21 (PMC8565517; doi:10.1128/mSphere.00850-21)
Supplement: TABLE S3 [file msphere.00850-21-st003.pdf]

Plasmid ID	Replicon (Incompatibility) type	Host strain ID	Host species by ANI	Antimicrobial resistance genes located on the plasmid[a]	Accession ID
pMTY12126_IncN+R	Multi-replicon IncN and IncR	TUM12126	*K. pneumoniae*	bla_{KPC-2}, bla_{OXA-9}, bla_{TEM-1}, *aph(4)-Ia, aac(6')-Ib-cr, aadA1, aac(3)-IV, aadA2, cmlA1*	AP024751
pMTY12127_IncN+R	Multi-replicon IncN and IncR	TUM12127	*K. pneumoniae*	bla_{KPC-2}, bla_{OXA-9}, bla_{TEM-1}, *aph(4)-Ia, aac(6')-Ib-cr, aadA1, aac(3)-IV, aadA2, cmlA1*	AP024754
pMTY12128_IncN+R	Multi-replicon IncN and IncR	TUM12128	*K. pneumoniae*	bla_{KPC-2}, bla_{OXA-9}, bla_{TEM-1}, *aph(4)-Ia, aac(6')-Ib-cr, aadA1, aac(3)-IV, aadA2, cmlA1*	AP024757
pMTY12128_ColRNAI	ColRNI	TUM12128	*K. pneumoniae*	bla_{KPC-2}, *aac(6')-Ib-cr*	AP024759
pMTY12129_IncN+R	Multi-replicon IncN and IncR	TUM12129	*K. pneumoniae*	bla_{KPC-2}, bla_{OXA-9}, bla_{TEM-1}, *aph(4)-Ia, aac(6')-Ib-cr, aadA1, aac(3)-IV, aadA2, cmlA1*	AP024761
pMTY12130_IncN+R	Multi-replicon IncN and IncR	TUM12130	*K. pneumoniae*	bla_{KPC-2}, bla_{OXA-9}, bla_{TEM-1}, *aph(4)-Ia, aac(6')-Ib-cr, aadA1, aac(3)-IV, aadA2, cmlA1*	AP024764
pMTY12131_IncN+R	Multi-replicon IncN and IncR	TUM12131	*K. pneumoniae*	bla_{KPC-2}, bla_{OXA-9}, bla_{TEM-1}, *aph(4)-Ia, aac(6')-Ib-cr, aadA1, aac(3)-IV, aadA2*	AP024767
pMTY12132_IncN+R	Multi-replicon IncN and IncR	TUM12132	*K. pneumoniae*	bla_{KPC-2}, bla_{OXA-9}, bla_{TEM-1}, *aph(4)-Ia, aac(6')-Ib-cr, aadA1, aac(3)-IV, aadA2, cmlA1*	AP024770
pMTY12133_IncN+R	Multi-replicon IncN and IncR	TUM12133	*K. pneumoniae*	bla_{KPC-2}, bla_{OXA-9}, bla_{TEM-1}, *aph(4)-Ia, aac(6')-Ib-cr, aadA1, aac(3)-IV, aadA2, cmlA1*	AP024773
pMTY12134_IncN+R	Multi-replicon IncN and IncR	TUM12134	*K. pneumoniae*	bla_{KPC-2}, bla_{OXA-9}, bla_{TEM-1}, *aph(4)-Ia, aac(6')-Ib-cr, aadA1, aac(3)-IV, aadA2, cmlA1*	AP024776
pMTY12134_ColRNAI	ColRNI	TUM12134	*K. pneumoniae*	bla_{KPC-2}, *aac(6')-Ib-cr*	AP024777
pMTY12135_IncN+R	Multi-replicon IncN and IncR	TUM12135	*K. pneumoniae*	bla_{KPC-2}, bla_{OXA-9}, bla_{TEM-1}, *aph(4)-Ia, aac(6')-Ib-cr, aadA1, aac(3)-IV, aadA2, cmlA1*	AP024779
pMTY12135_ColRNAI	ColRNI	TUM12135	*K. pneumoniae*	bla_{KPC-2}, *aac(6')-Ib-cr*	AP024780
pMTY12136_IncN+R	Multi-replicon IncN and IncR	TUM12136	*K. pneumoniae*	bla_{KPC-2}, bla_{OXA-9}, bla_{TEM-1}, *aph(4)-Ia, aac(6')-Ib-cr, aadA1, aac(3)-IV, aadA2, cmlA1*	AP024782
pMTY12137_IncN+R	Multi-replicon IncN and IncR	TUM12137	*K. pneumoniae*	bla_{KPC-2} (two copies), bla_{OXA-9}, bla_{TEM-1}, *aph(4)-Ia, aac(6')-Ib-cr, aadA1, aac(3)-IV, aadA2, cmlA1*	AP024786
pMTY12138_IncN+R	Multi-replicon IncN and IncR	TUM12138	*K. pneumoniae*	bla_{KPC-2}, bla_{OXA-9}, bla_{TEM-1}, *aph(4)-Ia, aac(6')-Ib-cr, aadA1, aac(3)-IV, aadA2, cmlA1*	AP024790
pMTY12139_IncN+R	Multi-replicon IncN and IncR	TUM12139	*K. pneumoniae*	bla_{KPC-2} (two copies), bla_{OXA-9}, bla_{TEM-1}, *aph(4)-Ia, aac(6')-Ib-cr, aadA1, aac(3)-IV, aadA2, cmlA1*	AP024793
pMTY12140_IncN+R	Multi-replicon IncN and IncR	TUM12140	*K. pneumoniae*	bla_{KPC-2} (two copies), bla_{OXA-9}, bla_{TEM-1}, *aph(4)-Ia, aac(6')-Ib-cr, aadA1, aac(3)-IV, aadA2, cmlA1*	AP024796
pMTY12147_ColRNAI	ColRNI	TUM12147	*C. europaeus*	bla_{KPC-2}, *aac(6')-Ib-cr*	BPMF01000050
pMTY12148_ColRNAI	ColRNI	TUM12148	*C. europaeus*	bla_{KPC-2}, *aac(6')-Ib-cr*	BPMG01000023
pMTY12149_ColRNAI	ColRNI	TUM12149	*M. morganii*	bla_{KPC-2}, *aac(6')-Ib-cr*	BPMH01000056
pMTY12150_ColRNAI	ColRNI	TUM12150	*M. morganii*	bla_{KPC-2}, *aac(6')-Ib-cr*	BPMI01000045
pMTY12151_ColRNAI	ColRNI	TUM12151	*M. morganii*	bla_{KPC-2}, *aac(6')-Ib-cr*	BPMJ01000028
