# Supplementary material for: Molecular Analysis of blaKPC-2-Harboring Plasmids: Tn4401a Interplasmid Transposition and Tn4401a-Carrying ColRNAI Plasmid Mobilization from Klebsiella pneumoniae to Citrobacter europaeus and Morganella morganii in a Single Patient
Source: mSphere. 2021 Nov 3;6(6):e00850-21. doi: 10.1128/mSphere.00850-21 (PMC8565517; doi:10.1128/mSphere.00850-21)
Supplement: TABLE S4 [file msphere.00850-21-st004.pdf]

Strain ID	GI number	Accession number	BioSample accession number	Isolation source	geographic location	Collection month, year
BWH22	583674870	JCNO01000001-JCNO01000023	SAMN02138591	urine	missing	June, 2012
BIDMC18A	583595570	JCNA01000001-JCNA01000015	SAMN02138642	urine	missing	May, 2012
BIDMC18C	550187172	AXLE01000001-AXLE01000006	SAMN02138644	urine	missing	November, 2010
MGH51	636346778	JMYV01000001-JMYV01000035	SAMN02581383	urine	Boston, USA	February, 2013
MGH71	636402080	JMZG01000001-JMZG01000035	SAMN02581244	urine	Boston, USA	June, 2013
MGH79	636422449	JMZL01000001-JMZL01000030	SAMN02581252	urine	Boston, USA	June, 2013
BAA-1705	NA	NZ_AOGQ01000001-NZ_AOGQ01000169	SAMN02471470	urine	missing	June, 2007
BIDMC60	635941492	JMWA01000001-JMWA01000026	SAMN02581281	peritoneal fluid	missing	August, 2013
MGH29	583735725	JCNY01000001-JCNY01000045	SAMN02138569	respiratory sample	missing	September, 2012
BIDMC1	583652499	JCNK01000001-JCNK01000015	SAMN02138620	bone tissue	missing	August, 2010
BIDMC12B	583616823	JCND01000001-JCND01000031	June, 2011	blood	missing	April, 2010
BIDMC16	550192397	AXLF01000001-AXLF01000006	SAMN02138639	blood	missing	November, 2009
BIDMC2A	583647214	JCNJ01000001-JCNJ01000012	SAMN02138621	blood	missing	May, 2010
BIDMC32	583595496	JCMY01000001-JCMY01000045	SAMN02138662	abscess	missing	Augsut, 2012
BIDMC5	583637337	JCNH01000001-JCNH01000027	SAMN02138625	blood	missing	July, 2008
BIDMC42a	583572189	JCMV01000001-JCMV01000022	SAMN02356579	blood	Boston, USA	December, 2012
BIDMC7A	583742431	JAPO01000001-JAPO01000029	SAMN02138627	urine	missing	April, 2012
BIDMC14	583604920	JCNB01000001-JCNB01000023	SAMN02138637	urine	missing	November, 2009
BWH45	635963057	JMWE01000001-JMWE01000020	SAMN02581255	tissue	Boston, USA	September, 2013
KPNIH1chr	NA	NZ_CP008827	SAMN01057611	groin	USA	June, 2011
